# Prevalence, population structure, distribution of serotypes, pilus islands and resistance genes among erythromycin-resistant colonizing and invasive *Streptococcus agalactiae* isolates recovered from pregnant and non-pregnant women in Isfahan, Iran

**DOI:** 10.1186/s12866-021-02186-2

**Published:** 2021-05-04

**Authors:** Tahereh Motallebirad, Hossein Fazeli, Ataollah Ghahiri, Dariush Shokri, Saba Jalalifar, Sharareh Moghim, Bahram Nasr Esfahani

**Affiliations:** 1 Department of Microbiology, School of Medicine, Isfahan University of Medical Sciences, Hezar-Jerib Street, Isfahan, Iran; 2 Department of Gynecology and Obstetrics, Al-Zahra university Hospital, Isfahan University of Medical Sciences, Isfahan, Iran; 3 Infectious disease and tropical medicine research center, Isfahan University of Medical Sciences, Isfahan, Iran

**Keywords:** *Streptococcus agalactiae*, Capsular genotyping, Pilus islands, Antibiotic susceptibility, MLST, Iran

## Abstract

**Background:**

The information on antibiotic resistance and molecular features of Group B *Streptococcus* (GBS) are essential for epidemiological purposes as well as vaccine development. Therefore, we aimed to assess the antimicrobial resistance profiles and molecular characteristics of GBS isolates in Isfahan, Iran. A total number of 72 colonizing and invasive GBS were collected from pregnant and non-pregnant women. The GBS isolates were analyzed for resistance profiles, capsular genotyping, and detection of PI-1, PI-2a, PI-2b, *hvgA*, *ermB, ermTR, lnuB* and, *mefA* genes. Besides, erythromycin-resistant strains were subjected to multilocus sequence typing (MLST).

**Results:**

The prevalence of colonizing and invasive GBS were 11 and 0.05%, respectively. The frequency of capsular serotypes was as follows: III (26.3%), Ia (20.83%), Ib and V (each 15.2%), IV (9.7%), II (8.3%), VII (2.7%), and VI (1.3%). Overall frequencies of PIs were as follows: PI-1, 37.5%, PI-1 + PI-2a, 30.5%, PI-1 + PI-2b, 29.1% and PI-2b, 2.7%. Two maternal colonizing GBS (2.6%) were *hvgA* positive and were belonged to ST-17/CPS-III/PI-1 + PI-2b lineage. Among 30(41.6%) erythromycin resistant GBS, 21 isolates (70%) harbored *ermB* gene, followed by *ermTR* (23.3%) and *mefA* (10%). One clindamycin-resistant isolate harbored the *lnuB* gene. MLST analysis revealed the following five clonal complexes (CCs) and nine STs: (CC-19/ST-335, ST-19, and ST-197), (CC-12/ST-43, ST-12), (CC-23/ST-163, ST-23), (CC-17/ST-17) and (CC-4/ST-16).

**Conclusion:**

The study shows an alarmingly high prevalence of erythromycin-resistant GBS in Iran. In addition, we report dissemination of ST-335/CPS-III clone associated with tetracycline and erythromycin resistance in our region. The distribution of capsular and pilus genotypes varies between invasive and colonizing GBS that could be helpful for vaccine development.

## Introduction

Group B *Streptococcus* (GBS, *Streptococcus agalactiae*), is regarded as one of the major causes of neonatal sepsis, pneumonia, and meningitis. This bacterium is increasingly associated with invasive infections in men and non-pregnant women and elderly patients with diabetes mellitus, cancer, renal dialysis, and other significant underlying diseases [1, 2].

The incidence of neonatal early-onset GBS infections has decreased since the use of intrapartum antibiotic prophylaxis (IAP) in GBS-colonized pregnant women [3, 4]. Penicillin is the drug of choice for intrapartum prophylaxis and treatment of GBS infections. However, reduced susceptibility to penicillin has been reported. Erythromycin, clindamycin, and levofloxacin are used as alternative therapeutic agents for β-lactam allergic patients [1, 5]. Unfortunately, the resistance of GBS to macrolides is increasing worldwide [6–8]. Previous studies have shown that the *ermB, ermTR, and mefA/E* genes are involved in the resistance to macrolides [9].

The capsular polysaccharide (CPS) is one of the most important virulence factors and a target helping vaccine development [10]. At present ten serotypes (Ia, Ib-IX) are identified in GBS strains based on unique serologic properties and distinct biochemical structures of sialic acid-rich CPS [11]. In addition to the capsule, GBS possesses a pilus-like structure mediating adhesion, immune evasion, and host cell invasion [12]. In GBS three pilus island (PI) alleles have been encoded including PI-1, PI-2a, and PI-2b. Another virulence factor in association with the invasive potential of the GBS strains for the host cells is Hyper virulent GBS adhesin (HvgA) encoded by the *hvgA* gene. This protein confers the ability of GBS strains to cross the intestinal and blood–brain barriers and causes the development of meningitis in infants born to colonized mothers [13]. CPS, and components of ancillary and backbone proteins of pilus, and HvgA were explored as the vaccine candidates and antibacterial therapeutic targets [14].

Several molecular typing methods have been applied to epidemiological studies, of which Multi-locus sequence typing (MLST) is a robust and convenient method that results in exchangeable data in different labs [15]. Analysis of MLST of the GBS population from different countries revealed that most of the human colonizing and clinical GBS isolates cluster into six Clonal Complexes (CCs) as follows: CC1, CC10, CC17, CC19, CC23, and CC26 [16]. In previous studies, STs 1 and 19 were considered as major important clones in terms of asymptomatic colonization and ST-23 was the most common ST related to the carriage and invasive GBS [17]. On the other hand, strains belonging to CC17 were the leading cause of many neonatal invasive infections [17].

Despite developed countries, there was no data on the prevalence of invasive GBS infection and clonal diversity of GBS strains among adults in Iran. Due to this issue in the current study, we aimed to assess the prevalence, distribution of virulence genes and erythromycin resistance-related genes, capsular typing of colonizing and invasive GBS strains isolated from pregnant and non-pregnant women and genetic diversity and clonality of the erythromycin-resistant population in Isfahan, Iran.

## Results

The prevalence of colonizing and invasive GBS was 11 and 0.05%, respectively. The age range of pregnant women was 18–40 and for non-pregnant women was 48–70 years old.

### Antibiotic susceptibility

The results of antimicrobial susceptibility patterns and D-zone test of 72 GBS isolates are presented in Table 1. All isolates were susceptible to penicillin, cefepime, ceftriaxone, cefotaxime, and vancomycin and showed high rates of resistance to tetracycline (90.2%), erythromycin (41.6%), and clindamycin (30.5%). The overall percentage of levofloxacin resistance was 9.7%.

**Table 1:** Antimicrobial susceptibility testing of 72 invasive and colonizing GBS

	Colonizing n (%)			Invasive n (%)		
Antibiotic	Susceptible	Intermediate	Resistant	Susceptible	Intermediate	Resistant
Penicillin	36 (100)	–	–	36 (100)	–	–
Cefepime	36 (100)	–	–	36 (100)	–	–
Ceftriaxone	36 (100)	–	–	36 (100)	–	–
Cefotaxime	36 (100)	–	–	36 (100)	–	–
Vancomycin	36 (100)	–	–	36 (100)	–	–
Tetracycline	2 (5.5)	–	34 (94.4)	5 (13.8)	–	31 (86.1)
Levofloxacin	29 (80.5)	5 (13.8)	2 (5.5)	27 (75%)	4 (11.1)	5 (13.8)
Clindamycin	14 (38.8)	11 (30.5)	11 (30.5)	19 (52.7)	6 (16.6)	11 (30.5)
Erythromycin	10 (27.7)	11 (30.5)	15 (41.6)	17 (47.2)	4 (11.1)	15 (41.6)
iMLSB^a^	5 (13.8)			4 (11.1)		
c MLSB ^b^	9 (25)			9 (25)		
M phenotype ^c^	1 (2.7)			2 (5.5)		
L phenotype ^d^	2 (5.5)			2 (5.5)		
MDR	10 (27.7)			6 (16.6)		
Total	36			36		

^a^ Inducible Macrolides, Lincosamides, and Streptogramin B (MLSB) phenotype with resistance to erythromycin and blunting of zone of inhibition of clindamycin

^b^ Constitutive MLSB with resistance to clindamycin and erythromycin

^c^ Resistant to erythromycin only but not clindamycin by efflux mechanism

^d^ Resistant to clindamycin only without blunting (without D shape)

Out of 72 GBS isolates, 16 (22.2%) isolates (10 colonizing and six invasive) had MDR pattern. Most MDR isolates were resistant to three antibiotics including tetracycline, erythromycin, and clindamycin. Five MDR isolates were also resistant to levofloxacin.

### Distribution of erythromycin resistance genes

Among the 30 erythromycin-resistant isolates, 18 (60%, Colonizing *n* = 9, Invasive *n* = 9), nine (30%, Colonizing *n* = 5, Invasive *n* = 4) and three (10%, Colonizing *n* = 1, Invasive *n* = 2), had cMLSB, iMLSB and M resistance phenotypes, respectively. A number of four isolates (5.5%, Colonizing *n* = 2, Invasive *n* = 2) showed L phenotype and were susceptible to erythromycin and resistant to clindamycin. The most prevalent resistance genes were *ermB* (21/ 30, 70%), followed by *ermTR* (7/ 30, 23.3%) and, *mefA* (3/ 30, 10%). All 18 GBS strains presenting the cMLSB phenotypes carried the *ermB* gene (*p* < 0.001) and one isolate was positive for both *ermB* and *ermTR* genes. The *ermTR* gene was associated with the iMLSB phenotype. From seven isolates harboring the *ermTR* gene, six isolates (85.7%) presented the iMLSB phenotype (*p* < 0.01). In addition, we found significant association between the *mefA* gene and the M phenotype, as, all three *mefA* positive isolates displayed the M phenotype (3 /3, 100%, *p* < 0.05) (Table 2). Based on the our results, methylation of the antibiotic binding site of 23S rRNA due to methylases encoded by the *erm* genes was the main mechanism of resistance to erythromycin (90%). The results of our study revealed an association between the resistance phenotype/ genotype and certain serotypes. A rate of 83.3% (*n* = 5 / 6) of the resistant serotype Ia strains were belonged to CPS-Ia/ cMLSB/ *ermB*, all resistant serotypes Ib and V strains were belonged to cMLSB/*ermB* profile (*n* = 6 / 6, 100%) and 71.4% (*n* = 5 / 7) of the resistant serotype III strains were CPS-III/ iMLSB/ *ermTR* (*p* < 0.05) (Table 3).

**Table 2:** Distribution of CCs, STs, PIs, serotypes and antibiotic resistance pattern among erythromycin-resistant GBS

Strain name	GBS type	Isolation source	CPS type	Pilus Island type	Resistance pattern	Erythromycin resistance phenotype	Resistance genes	ST	CC
MS 1040	Colonizing	RVS^e^	III	PI-1	E- C-T	cMLSB	*ermB*	335	19
MS 983	Invasive	Blood	V	PI-1 + PI-2b	E ^a^-C ^b^-T ^c^-LEV ^d^	cMLSB	*ermB*	335	
MS 422	Invasive	Blood	III	PI-1 + PI-2b	E- T- LEV	iMLSB	*ermTR*	335	
MS 34	Invasive	Joint fluid	III	PI-1 + PI-2a	E- T	iMLSB	*ermTR*	335	
MS 302	Invasive	Blood	III	PI-1 + PI-2a	E- T	M	*mefA*	335	
MS 161	Invasive	Blood	III	PI-1 + PI-2b	E- T	M	*mefA*	335	
MS 655	Colonizing	RVS	V	PI-1	E- C-T	cMLSB	*ermB*	335	
MS 5	Colonizing	RVS	III	PI-1 + PI-2b	E- T	iMLSB	*ermB*	335	
MS 334	Invasive	Blood	V	PI-1 + PI-2a	E- C- T- LEV	cMLSB	*ermTR + ermB*	335	
MSMOT	Invasive	Blood	III	PI-1	E- C	cMLSB	*ermB*	335	
MS 135	Invasive	Blood	IV	PI-1 + PI-2b	E- C	cMLSB	*ermB*	197	
MS 101	Colonizing	RVS	III	PI-1	E -T	iMLSB	*ermTR*	19	
MS 651	Invasive	Blood	Ib	PI-1 + PI-2b	E- C	cMLSB	*ermB*	19	
MS 300	Invasive	Blood	VII	PI-1 + PI-2a	E- C- T- LEV	cMLSB	*ermB*	19	
MS 102	Colonizing	RVS	Ib	PI-1 + PI-2b	E- LEV	iMLSB	*ermB*	43	12
MS 663	Colonizing	RVS	II	PI-1	E- C- T- LEV	cMLSB	*ermB*	43	
MS 40	Colonizing	RVS	Ia	PI-1 + PI-2a	E- C- T	cMLSB	*ermB*	43	
MS 68	Colonizing	RVS	IV	PI-1	E- C- T	cMLSB	*ermB*	43	
MS 949	Invasive	Peritoneal fluid	VI	PI-1	E	iMLSB	*ermTR*	43	
MS 278	Invasive	Plural fluid	III	PI-1 + PI-2b	E- T	iMLSB	*ermTR*	12	
MS 865	Invasive	Joint fluid	Ib	PI-1	E- C-T	cMLSB	*ermB*	12	
MS 816	Invasive	Blood	III	PI-1	E- C- T	cMLSB	*ermB*	12	
MS 305	Colonizing	RVS	Ia	PI-2b	E- T	M	*mefA*	12	
MS 358	Invasive	Blood	Ia	PI-2b	E- C	cMLSB	*ermB*	16	4
MS 76	Colonizing	RVS	III	PI-1 + PI-2a	E- T	iMLSB	*ermB*	163	23
MS 326	Colonizing	RVS	Ia	PI-1	E- C- T	cMLSB	*ermB*	163	
MS 177	Colonizing	RVS	Ia	PI-1	E- C- T	cMLSB	*ermB*	163	
MS 120	Colonizing	RVS	Ia	PI-1	E- C- T	cMLSB	*ermB*	23	
MS 100	Colonizing	RVS	III	PI-1 + PI-2b	E	iMLSB	*ermTR*	17	17
MS 136	Colonizing	RVS	III	PI-1 + PI-2b	E- C	cMLSB	*ermB*	17	

^a^. Erythromycin, ^b^. Clindamycin, ^c^. Tetracycline, ^d^. Levofloxacin. ^e^Recto-vaginal swab

**Table 3:** Distribution of resistance phenotypes and genotypes across serotypes among erythromycin-resistant GBS

	Ia	Ib	II	III	IV	V	VI	VII	Total
N(%)of resistant strain	6 (20)	3 (10)	1 (3.3)	13 (43.3)	2 (6.6)	3 (10)	1 (3.3)	1 (3.3)	30 (100)
*ermB*	5 (23.8)	3 (14.2)	1 (4.7)	6 (28.5)	2 (9.5)	3 (14.2)	–	1 (2.8)	21 (70)
*ermTR*	–	–	–	5 (71.4)	–	1 (14.2)	1 (14.2)	–	7 (23.3)
*mefA*	1 (33.3)	–	–	2 (66.6)	–	–	–	–	3 (10)
cMLSB	5	2	1	4	2	3	–	1	18 (60)
iMLSB	–	1	–	7	–	–	1	–	9 (30)
M	1	–	–	2	–	–	–	–	3 (10)

In the present study, 22 isolates were clindamycin-resistant. Of them, 18 isolates (81.8%) had cMLSB phenotype and four isolates exhibited the L phenotype (18.2%). All these isolates had negative results for the detection of the *ermB, ermTR* and *mefA* genes. However, one isolate was positive for the *lnuB* gene. Three isolates had negative results for all of genes.

### Distribution of CPS, PIs and *hvgA* genes

A total of eight capsular types were identified and all of GBS were typeable. The frequency of serotypes was as follows: III (26.3%), Ia (20.83%), Ib and V (each 15.2%), IV (9.7%), II (8.3%), VII (2.7%), and VI (1.3%). VIII and IX serotypes were not detected in this study (Table 4). In total, CPS III accounted for 30.5% of the strains causing invasive disease in women. For colonized GBS strains, type Ia (30.5%) was predominant.

**Table 4:** Distribution of capsular genotypes and pilus islands of 72 GBS isolates

Capsular type	Colonizing	Invasive	Total
Ia	11 (30.5)	4 (11.1)	15 (20.8)
Ib	6 (16.6)	5 (13.8)	11 (15.2)
II	2 (5.5)	4 (11.1)	6 (8.3)
III	8 (22.2)	11 (30.5)	19 (26.3)
IV	3 (8.3)	4 (11.1)	7 (9.7)
V	6 (16.6)	5 (13.8)	11 (15.2)
VI	–	1 (2.7)	1 (1.3)
VII	–	2 (5.5)	2 (2.7)
VIII	–	–	–
**Pilus type**			
PI-1	19 (52.7)	8 (22.2)	27 (37.5)
PI- 1 + 2a	7 (19.4)	15 (41.6)	22 (30.5)
PI-1 + 2b	9 (25)	12 (33.3)	21 (29.1)
PI-2b	1 (2.7)	1 (2.7)	2 (2.7)

**Fig. 1:**
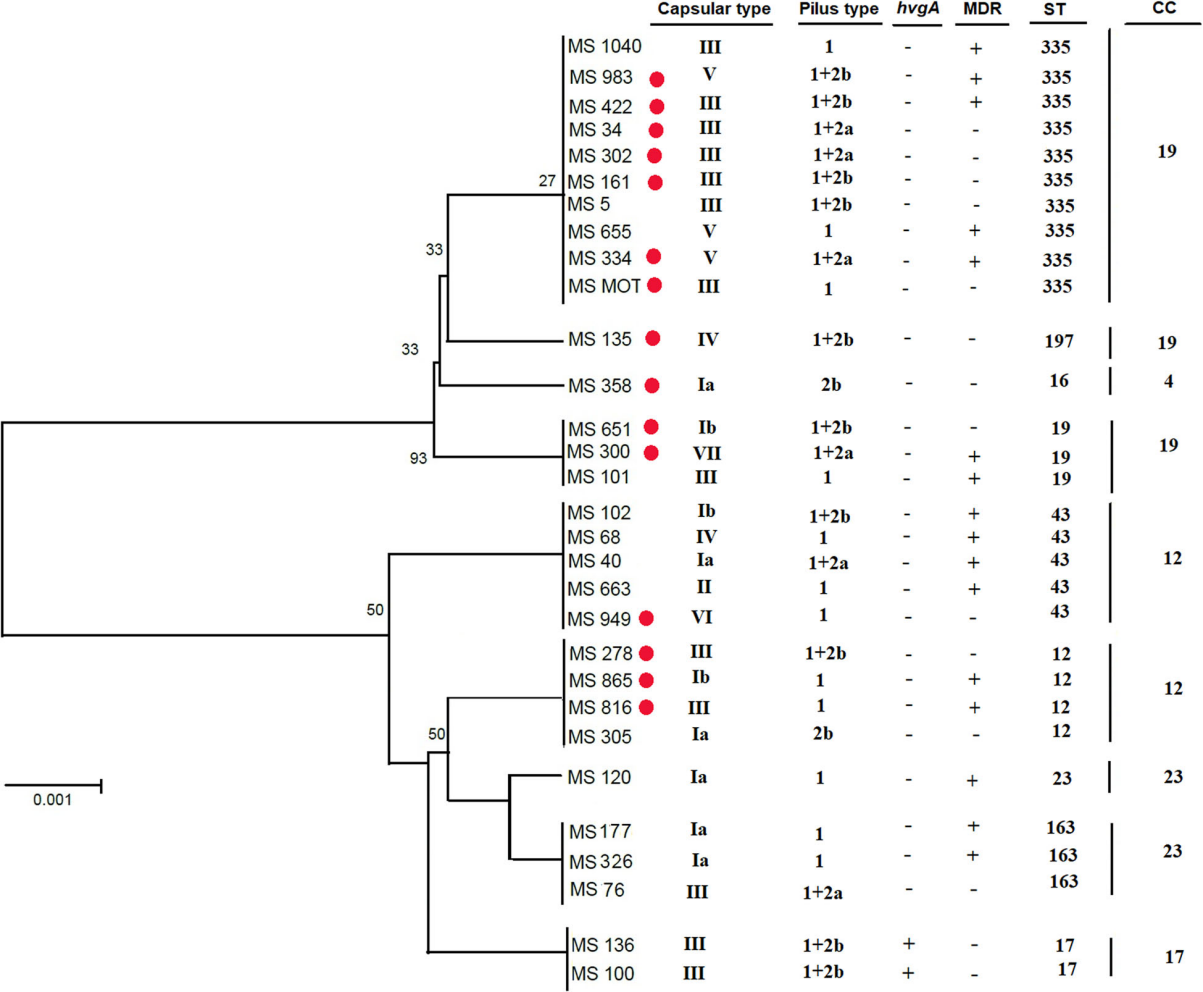
Phylogenic tree of concatenated sequences from the 30 erythromycin-resistant invasive and colonizing GBS strains. Each isolate was presented by the type of CPS, PIs, *hvgA* gene, MDR pattern, STs and CCs. The bullets identify invasive GBS isolates. Vertical bars on the far right identify groups of isolates with the same STs

At least one PI was detected in all of the isolates. Results revealed that PI-1 was detected in all GBS strains except two isolates. Colonizing GBS strains were significantly more likely to have PI-1 alone (19 isolates, 52.7%). PI-1 + PI-2a was detected in 15 (41.6%) invasive strains (Table 4). Statistical analysis of PIs distribution among colonizing and invasive strains showed a significant association between PI-1 and maternal colonizing GBS and PI-1 + PI-2a and invasive strains (*p* < 0.04). Among erythromycin-resistant GBS, the most frequent serotype and Pilus Island were CPS III (13/ 30, 43.3%) and PI-1 (12/ 30, 40%), respectively (Table 2). Two colonizing isolates (2.7%) were positive for the *hvgA* gene. These isolates were belonged to ST-17/ CPS-III lineage and harbored PI-1 + PI-2b. (Fig. 1).

### MLST

**Fig. 2:**
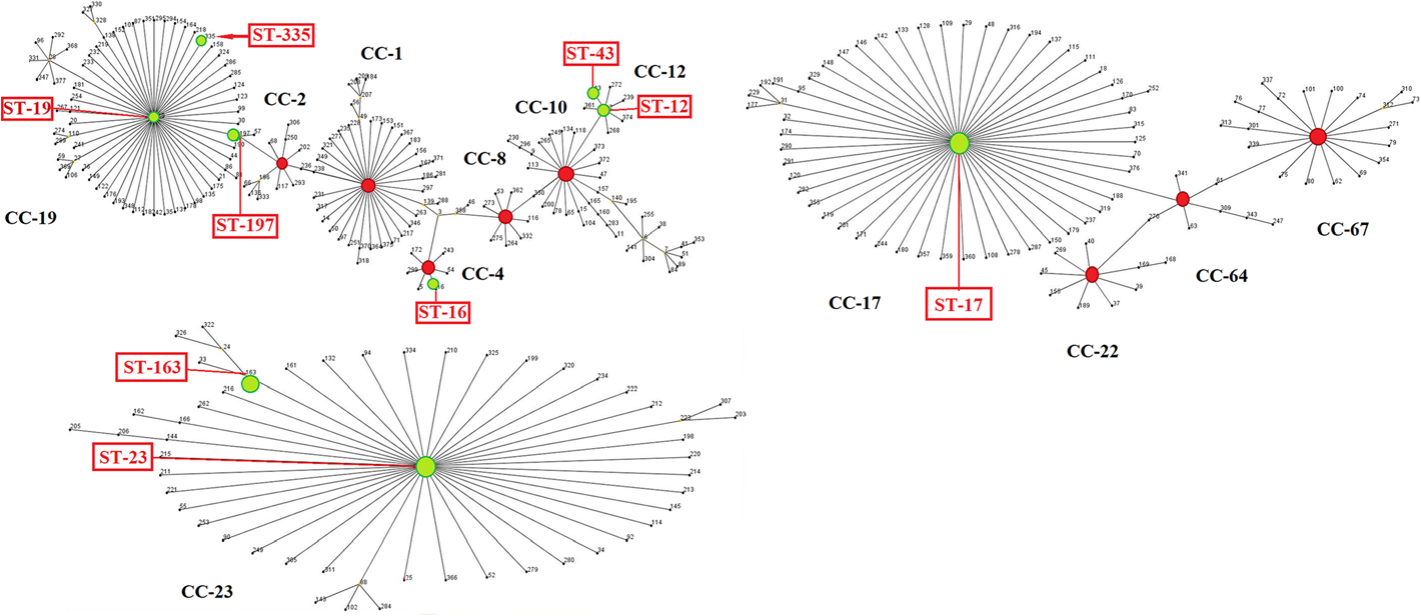
eBURST analysis of GBS strains of this study using all STs available in the MLST database. Red bullet are group founder and green bullets are STs in the current study. Boxes pointed by arrows indicate the STs detected in this study

Analysis of 30 erythromycin-resistant GBS demonstrated the existence of different genetic lineages among strains expressing the same capsular genotype, PIs, and erythromycin- resistance genes. We identified nine different STs belonging to five CCs. CC-19 contained the highest number of erythromycin-resistant GBS identified in this study followed by CC-12, CC-23, CC-17, and CC-4 (Fig. 2, Table 2). The most prevalent ST was ST-335 (10, 33.3%) followed by ST-43 (5, 16.6%), ST-12 (4, 13.3%), ST-163 and ST-19 (each 3, 10%), ST-17 (2, 6.6%), ST-23, ST-197 and ST-16 (each 1, 3.3%) (Fig. 1). Phylogenic analysis of our STs in each CCs revealed a high similarity of genome composition among STs (Fig. 1). ST-335/ CPS-III was the dominant clone (7/ 30, 23.3%).

With regard to the source of isolates, the most prevalent ST and CPS among the invasive strains were ST-335 and CPS III, whereas in colonized strains in pregnant mothers were ST-43 and CPS Ia. Colonizing GBS had relatively higher diversity than invasive ones and were distributed between all five CCs, whereas invasive strains were not detected in CC-17 and CC-23 (Simpson index of diversity = 0.78 for colonizing GBS and 0.51 for invasive ones).

To facilitate comparison of the clonal distribution of macrolide resistance, an analysis of the combination of MLST-type and MLSB resistance gene has often been used as a rough marker for resistant clones in GBS. Our results revealed that all strains belonged to CC-23 and CC-4 carrying the *ermB* gene. While, in CC-19, nine strains (64.2%), in CC-12 six strains (66.6%), and in CC-17 one strain harbored the *ermB* gene. A number of 17 different lineages were identified in resistant GBS isolates that ST-335/ *ermB* (*n* = 6) followed by ST-43/*ermB* (*n* = 4) were dominant.

## Discussion

In the present study, we sought to examine the prevalence of colonizing and invasive GBS in Iran, with a particular emphasis on capsular genotyping, determination of virulence genes profile, and antimicrobial resistance. Moreover, we used MLST to characterize erythromycin-resistant GBS and their associated resistance genes, as well as explore phylogenetic relationships and clonality in the resistant bacterial population.

Colonization carries the risk of invasive GBS disease in babies (before or after birth), and pregnant/postnatal women. Burden of GBS invasive disease was estimated to be 319,000 infants in 2015, resulting in 90,000 deaths, at least 57,000 fetal infections/stillbirth, and up to 3.5 million preterm births [18]. The prevalence of maternal colonization in the current study was 11%. The recent meta-analysis by Yektakooshali et al. showed the overall prevalence of maternal colonization in our country was 13.5% [19]. Worldwide around 18% of pregnant women are colonized by GBS (totaling over 21 million women each year) with a low rate of 8% in South Korea and 35% in South Africa [20].

In this study, the estimated incidence of invasive infections in non-pregnant women was 0.4 cases per 100,000. In England, Lamangi et al. found out of 21,376 clinical isolates, 62.5% belonged to invasive infection of non-pregnant adults and the incidence was 2.9 cases per 100,000 [21]. In Réunion Island, the incidence of invasive infections in non-pregnant adults was twofold that usually reported and 10.1 cases per 100,000 [22]. Another study in Australia showed out of 663 invasive GBS, 58.8% were collected from non-pregnant women [23]. GBS disease in the adults and elderly was not included in previous burden estimates, but is increasingly recognized public health issue, causing morbidity and mortality [24].

We identified resistance to erythromycin in a substantial number of the GBS strains (41.6%). The recent meta-analysis by Khademi et al. showed the overall rate of erythromycin resistance in Iran was 21%. This prevalence is lower than that reported rate in our study. In other parts of the world different results were reported; 20.7% in Spain [25], 40% in France [26], 41.7% in Hungaria [27], and 78.9% in China [28]. Regarding erythromycin resistance phenotype, our isolates had cMLSB phenotype in 60% of cases followed by iMLSB (30%) and M (10%). cMLSB phenotype was strongly related to the *ermB* gene, whereas most the iMLSB-GBS isolates harbored the *ermTR* gene and the M phenotype was associated with the *mefA* gene. In France, 46% of resistant isolates, had cMLSB phenotype/ *ermB* gene and 24.3% were M phenotype that all harbored *mefA* gene [26]. In another study, the cMLSB phenotype was related to the *ermB* gene, whereas the M phenotype was developed by the *ermA* gene and the iMLSB phenotype was dominant [29]. The efflux pump encoded by the *mefA* gene unable to pump out clindamycin and other lincosamides even in the presence of erythromycin. So strains harboring *mefA* gene cannot develop MLSB or L phenotype [30]. These data highlighted that the *erm* family genes are the predominant determinants responsible for resistance to erythromycin among GBS isolates [31, 32].

As aforementioned, 22 (30.5%) clindamycin-resistant GBS were identified in the present study. This prevalence is higher than that reported rate in Ethiopia (17.5%) [7], Spain (17.5%) [25] and UK (27%) [33], whereas it is lower than a report in China (76.8%) [28]. A meta-analysis performed in Iran notified that 26.8% of GBS strains obtained from pregnant women were detected as clindamycin-resistant [34]. Most of the clindamycin-resistant isolates (81.8%) showed cMLSB phenotype through the *ermB* gene. However, four isolates were L phenotype. One isolate was positive for the *lnuB* gene. Clindamycin resistance in three of our strains was not associated with either the *mefA, ermB* or *ermTR*, and *lnuB* genes. Such resistance in beta-hemolytic streptococci may be related to other resistance-associated genes such as *lnuA* or *erm* and *lsa* genes family or mutations in ribosomal proteins [35, 36]. A previously studied experiment from Norway noted that all GBS isolates with L phenotype harbored the *lsaC* gene, on the other hand, this gene was not present in any of the tested isolates with MLSB or M phenotype [37]. Other studies from Korea and the USA, revealed that the *lnuB* gene was responsible for L phenotype among GBS isolates [36, 38]. In China, among L phenotype isolates the *lnuB* gene was located in the multi-resistance gene cluster possibly acts as a composite transposon flanked by IS1216 and as a vehicle for the dissemination of multidrug resistance among GBS [39].

According to the literature review, a significant association between antibiotic resistance and certain serotypes or STs has been found [32, 40]. Although the small sample size of isolates is a limitation, colonizing and invasive erythromycin-resistant GBS isolates were distributed across multiple CCs. The majority of our erythromycin-resistant isolates were *ermB* positive (70%) and belonged to CC-19/ ST-335/ CPS-III (23.3%), a one-allele variant of ST-19. The emergence of this lineage has previously been documented in several other countries. Such clonal distribution can have a rapid and major impact on resistance rates. ST-335 is an epidemic invasive GBS disease strain in Japan and is dominantly correlated with serotype III and erythromycin resistance (via *mefA/E* gene) [41]. In another Asian country, South Korea, ST-335/III/ *ermA* was in relation to invasive infections in infants and erythromycin resistance [42]. In Serbia ST-1/CPS-V and ST-23/ CPS-III were dominant clones among erythromycin- resistant strains [43]. Binghuai et al. found a high prevalence of ST-17/ CPS-III among erythromycin-resistant isolates [28]. In Canada, Teatero et al. reported ST-1/CPS-V and ST-459/CPS-IV as dominant clones among macrolide -resistant strains [44]. Our findings are in compliment with former studies in East Asian countries, South Korea and Japan. The results of the current study revealed a high prevalence of the ST-335/*ermB* (35.2%) and ST-43/ *ermB* (23.5%) lineages among 17 different clones in our region. Previous studies showed the multiclonal spread of the *erm* genes predominantly in ST-17/ *ermB*, ST-1/*ermB*, ST-23/*ermB* and ST-12/*ermB* clones [37, 45, 46]. Our data showed that the erythromycin resistance genes were broadly distributed among different serotypes and STs, suggesting that the presence of these resistance genes among GBS populations may represent a consequence of multiple horizontal gene transfer events. Antimicrobial resistance spreading by conjugative transfer is less affected by herd immunity in the host population, and thus likely more challenging to combat [47]. In a study in Norway Whole-genome sequencing of resistant beta-hemolytic streptococci revealed that the mobile genetic elements harboring the resistance determinants showed remarkable intra and inter-species similarities, suggesting dissemination of antimicrobial resistance predominantly through conjugative transfer rather than clonal expansion of resistant strains in GBS [37]. The emergence of dominant clones continuously renders the phylogenetic landscape and is reflected in the fluctuating course of the resistance rates. The continued emergence of ST-335/ CPS-III lineage or other macrolide-resistant GBS would have obvious serious implications both for the formulation of treatment and prophylaxis recommendations and for the development of candidate GBS vaccines [37].

We found prevalent CPS were III, Ia, Ib, and, V. These findings are in agreement with previous studies in Portugal and China [48, 49]. Nonetheless, in Japan VI and VIII, in Egypt V, in Mexico Ia and in the USA and Europe Ia, Ib, II, III, and, V were the most common serotypes [17]. With respect to the source of GBS isolates, our results showed a high prevalence of CPS Ia in maternal colonization compared to invasive ones. A previous meta-analysis found CPS Ia to be the most frequent serotype contributing to maternal colonization and EOD in South America, United States, United Kingdom, and France. In contrast, in Asia, serotype Ib is well known for its association with Maternal colonization and EOD [1]. Other studies have described that the most common maternal colonizing GBS serotypes globally were serotype III (25%) and Ia (19%) [24]. The distribution of serotypes among our invasive GBS was different and serotype III was more common. A meta-analysis by Bianchi et al. showed that among non-pregnant adults with invasive infections serotype V was the most common with 25% followed by serotype Ia with 23% and serotype III with 11% [24]. In contrast, in African countries such as Egypt, Gabon and, Gambia and in North America serotype V was frequently recovered from maternal colonization [1, 17]. In south-eastern Asia, serotypes VI to IX have a higher presence than in other regions, representing 31% of adults’ invasive infections [24]. To a lesser extent, we found that all serotypes VI and VII isolates (4%) were isolated from non-pregnant women with invasive infection.

Statistical analysis of the current study revealed a significant association between PIs types and GBS sources. However, we found no association between erythromycin resistance and PIs distribution. Based on the present study, a significant relationship was found between PI-1 with maternal colonization and PI-1 + PI-2a with invasive infection. Invasive isolates combination of PI-1 with one of the PI-2 variants was accounted for 75% of cases. Former studies confirmed a significant association between the distribution of PI types across phylogenetic lineages and sources [50, 51]. Data about the distribution of PIs in our country is limited. Only two studies showed PI-1 + PI-2a as predominant PIs among invasive and non-invasive GBS isolated from adults [29, 52]. We found that the distribution of PIs was consistent with the studies performed in South Africa [51] and China [28] but similar to the results of a study in Portugal [53].

Unfortunately, there is no detailed information about the genetic population structure of GBS isolates among adults in Iran. Only one study on the colonization of GBS in throat and external ear canals of neonates’ population revealed three clonal complexes with the predominance of CC-19 followed by CC-10 and CC-1 and eight STs [54]. The population structure of our isolates is somehow similar to other parts of the world, suggesting in our region, like other countries, few common human-specific clones have spread to all regions of the world [15, 16, 50, 55]. However, we also report CC-4/ST-16/ CPS Ia/ *ermB*^*+*^ as a rare erythromycin resistance- associated clone which can cause invasive infection in humans.

We found two strains belonging to CC-17/ ST-17/ CPS III/ PI-1+ PI-2b/ *hvgA*+ clone from two colonized mothers. The *hvgA* gene was distributed among several clones such as CC-1/ CPS-V, CC-17/ CPS-III, CC-17/ CPS-IV [44]. These clones were in association with maternal colonization or invasive infections in neonates and adults [44]. The *hvgA*-positive GBS isolated from infants were belonged to serotype III, while in adults and elderly were serotypes III, IV and, V [44]. ST-17/ CPS-III/ *hvgA* clone was most frequently reported from the neonates with invasive infections especially meningitis, whereas is rare among adults [11, 13]. Colonized mothers with ST-17/CPS III/ *hvgA*^+^ clone, can act as reservoirs who can transmit the GBS to their babies during labor or through breast milk that may lead to EOD or LOD [28, 56].

## Conclusion

This study provided the first information on the population structure and genetic diversity of erythromycin-resistant colonizing and invasive GBS strains among adults in Iran. Most the erythromycin-resistant strains have been linked with CC19. ST-335/CPS-III was the most common circulating clone in the resistant population of women in our region. The results of this study confirm that the use of penicillin as a drug of choice for prophylaxis and treatment of GBS infections in Iran is appropriate. Erythromycin and clindamycin should no longer be relied upon as an alternative agent for prophylaxis and treatment of GBS infections in Iran without susceptibility testing. The relatively high level of erythromycin resistance detected by us is worrisome, as, in the case of penicillin-allergic patients, clindamycin is the drug of choice for treatment and intrapartum antibiotic prophylaxis according to the CDC guidelines [57]. If the isolate is resistant to clindamycin or D-test positive, then vancomycin is suggested. These results indicated the need for continuous monitoring of antimicrobial resistance for better management of preventive strategies in pregnant women and treatment of invasive infections.

## Materials and methods

### Bacterial isolates

From July 2016 to September 2018, in a cross-sectional study, a number of 72 GBS isolates including 36 colonizing and 36 invasive GBS strains were collected from pregnant and non-pregnant women who were referred to a teaching hospital in Isfahan, Iran. The Ethics Committee of Isfahan University of Medical Sciences approved this study, and the study was performed following the approved guideline (IR.MUI.REC.1396.3.125). The cases studied in this survey were healthy pregnant women being in the 35th–37th week of their gestation and non-pregnant women with invasive infections. Pregnant women were excluded if they had recently been treated for vaginal inflammation or infection and had used antibiotics within the last 4 weeks. According to Centers for Disease Control and Prevention (CDC) guidelines, after providing informed consent by participants, a recto-vaginal specimen was collected using two cotton swabs from 327 pregnant women between the 35th–37th week of gestation from the middle third region of the vagina and in the rectum through the anal sphincter. Samples were placed in Amie’s transport medium (Merck, Germany) and transferred to the laboratory. For isolation and characterization of GBS, the samples were processed based on standard isolation procedures [57]. In summary, the swabs were put in a tube containing 3 ml of Todd-Hewitt broth (Hi-Media, India) supplemented with nalidixic acid (15 μg/mL), gentamicin (8 μg/mL) and 5% sheep blood. After overnight incubation, aliquots of broth media were then inoculated onto Trypticase Soy Agar (TSA) (Merck, Germany) with 5% sheep blood and incubated overnight at 37 °C [57]. Typical colonies of GBS were confirmed with conventional and molecular assays and isolates were stored at − 70 °C in Trypticase Soy Broth with 20% glycerol [44]. During the study period, from 88,000 non-pregnant women referred to the clinical laboratory of the hospital, a total number of 36 invasive GBS which had been isolated from sterile body fluids, included in the study.

### Antibiotic susceptibility test

Based on the Clinical and Laboratory Standards Institute (CLSI) (2018 edition) [58], disc diffusion method was utilized to determine the antimicrobial susceptibility patterns to nine antibiotics including clindamycin (2 μg), vancomycin (30 μg), erythromycin (15 μg), penicillin (10 μg), tetracycline (30 μg), cefepime (30 μg), cefotaxime (30 μg), ceftriaxone (30 μg), and levofloxacin (5 μg); (MAST, Merseyside, UK). Multidrug resistance (MDR) was defined as the resistance of different classes tested in this study. The detection of erythromycin resistance phenotypes (constitutive and inducible MLSB (Macrolide-Lincosamide-Streptogramin B), M and L phenotypes) was done using the D-zone test as previously described [59].

### DNA extraction

Genomic DNAs of GBS isolates were extracted using a simple boiling method. In summary, a loopful of bacterial biomass was suspended in 300 μl of TSE buffer [100 mM Tris hydrochloride (pH 7.5), 25 mM EDTA, 1% SDS], and the suspension was heated at 95 °C for 20 min and centrifuged at 10000 g for 10 min. The supernatant was taken as DNA lysate and was kept at − 20 °C for the molecular assay [60].

### Molecular CPS genotyping

Molecular serotyping was performed using nine primer pairs for the detection of Ia-VIII capsular types as described previously [61]. A primer pair (*dltS*-F and *dltS*-R) targeting the GBS-specific *dltS* gene was also included as an internal positive control [61].

### Virulence gene detection

The distribution of PIs (PI-1, PI-2a, and PI-2b) and *hvgA* genes was determined using the specific primers and multiplex PCR reaction as already reported [62, 63].

### Detection of resistance genes

In order to determine specific erythromycin resistance genes, the PCR amplification of *ermTR*, *ermB*, and *mefA* genes was conducted for all 30 erythromycin-resistant GBS isolates [9]. Specific *dltS* gene was used as a positive internal control. In addition, all L phenotype clindamycin-resistant GBS isolates were surveyed for *ermB*, *ermTR, mefA* and *lnuB* genes using specific primers and PCR condition as previously described [64].

### MLST

A total of 30 erythromycin-resistant strains (15 colonizing and 15 invasive isolates) were subjected to MLST. Primers and PCR conditions for amplification of seven housekeeping genes of GBS were implemented as described by Manning [55]. MLST analysis was performed by submitting the obtained DNA sequences to the online MLST database available at http://mlst.net.http://pubmlst.org. Clonal complexes (CCs) were determined using the program e-BURST v3 based on related STs (https://www.phyloviz.net/goeburst/). The phylogenetic tree was inferred by using MEGA 8 software [65] according to the UPGMA method with high bootstrapping value to investigate the relatedness of STs with antibiotic resistance, PIs genes, and capsular serotypes.

### Statistical analysis

The SPSS Statistics (IBM SPSS Statistics for Windows, V.20.) were used for statistical analysis. To assess if any clonal complex, capsular serotype, and PIs are associated with colonizing or invasive isolates, the distribution of each clonal complex was examined against random distribution by utilizing the Pearson chi-square test. Association between clonal complex and capsular serotype or PIs distributions was assessed by applying Fisher’s exact test. Differences were considered statistically significant at *p* < 0.05.

Simpson’s index of diversity was used to quantify the clonal diversity between the colonizing and invasive isolates studied.

## Data Availability

Data are available as request to the corresponding author.

## Abbreviations

GBS: Group B Streptococci
PI: Pilus Island
CPS: Capsular Poly Saccharide
ST: Sequence Type
CC: Clonal Complex
MLST: Multi-Locus Sequence Typing
RVS: Recto-vaginal swab
MDR: Multi-Drug Resistant
